# Tautomerase Activity-Lacking of the Macrophage Migration Inhibitory Factor Alleviates the Inflammation and Insulin Tolerance in High Fat Diet-Induced Obese Mice

**DOI:** 10.3389/fendo.2020.00134

**Published:** 2020-03-20

**Authors:** Yan-Hong Li, Ke Wen, Ling-Ling Zhu, Sheng-Kai Lv, Qing Cao, Qian Li, Libin Deng, Tingtao Chen, Xiaolei Wang, Ke-Yu Deng, Ling-Fang Wang, Hong-Bo Xin

**Affiliations:** ^1^National Engineering Research Center for Bioengineering Drugs and the Technologies, Institute of Translational Medicine, The First Affiliated Hospital, Nanchang University, Nanchang, China; ^2^Basic Medical School, Nanchang University, Nanchang, China

**Keywords:** macrophage migration inhibitory factor, obesity, inflammation, insulin resistance, apoptosis

## Abstract

Macrophage migration inhibitory factor (MIF) has multiple intrinsic enzymatic activities of the dopachrome/phenylpyruvate tautomerase and thiol protein oxidoreductase, and plays an important role in the development of obesity as a pro-inflammatory cytokine. However, which enzymatic activity of MIF is responsible for regulating in obesity are still unknown. In the present study, we investigated the roles of the tautomerase of MIF in high fat diet (HFD)-induced obesity using MIF tautomerase activity-lacking (MIF^P1G/P1G^) mice. Our results showed that the serum MIF and the expression of MIF in adipose tissue were increased in HFD-treated mice compared with normal diet fed mice. The bodyweights were significantly reduced in MIF^P1G/P1G^ mice compared with WT mice fed with HFD. The sizes of adipocytes were smaller in MIF^P1G/P1G^ mice compared with WT mice fed with HFD using haematoxylin and eosin (H&E) staining. In addition, the MIF^P1G/P1G^ mice reduced the macrophage infiltration, seen as the decreases of the expression of inflammatory factors such as F4/80, IL-1β, TNFα, MCP1, and IL-6. The glucose tolerance tests (GTT) and insulin tolerance tests (ITT) assays showed that the glucose tolerance and insulin resistance were markedly improved, and the expressions of IRS and PPARγ were upregulated in adipose tissue from MIF^P1G/P1G^ mice fed with HFD. Furthermore, we observed that the expressions of Bax, a pro-apoptotic protein, and the cleaved caspase 3-positive cells in white tissues were decreased and the ratio of Bcl2/Bax was increased in MIF^P1G/P1G^ mice compared with WT mice. Taken together, our results demonstrated that the tautomerase activity-lacking of MIF significantly alleviated the HFD-induced obesity and adipose tissue inflammation, and improved insulin resistance in MIF^P1G/P1G^ mice.

## Introduction

Due to consumption of a high fat diet (HFD) and the change of life style, the incidence of obesity has been substantially increased over the past 40 years, from less than 1% in 1975 to 6–8% in 2016 (1). Obesity belongs to chronic metabolic disease caused by genetic and environmental factors, characterized by abnormal or excessive fat accumulation, especially in adipose tissue (2). When energy intake surpasses energy expenditure over time, obesity is developed. Numerous studies indicated that obesity could greatly increase the risk of the development of cardiovascular diseases, cancer, Alzheimer's disease, and diabetes mellitus (3–6).

It has been reported that HFD-induced obesity was associated with a chronic state of low-grade inflammation, which then engendered insulin resistance (7). Adipose tissue not only is the major site of energy storage, but also is an active endocrine organ (8). Studies showed that adipocytes and the infiltrated macrophages in adipose tissue produced a lot of pro-inflammatory mediators including macrophage migration inhibitory factor (MIF), IL-1β, leptin and adiponectin and so on (9, 10). All these cytokines and adipokines seriously affected the differentiation and functions of adipocytes through interfering with insulin signaling and intracellular stress pathways (11, 12). Moreover, many studies showed that chronic inflammation in adipose tissue can lead to insulin resistance (13–15). It has been reported that the insulin resistance was improved by reducing inflammation in adipose tissues (16, 17). In addition, adipose tissue also plays an important role in glucose homoeostasis. It was reported that the expression of GLUT4 was decreased in adipose tissue and then the glucose uptake was reduced in insulin resistance status (18). Therefore, to inhibit adipose tissue inflammation may provide a therapeutic strategy for improving insulin resistance in obese people.

MIF was originally identified as a T lymphocyte cytokine for inhibiting the random migration of macrophages *in vitro* in 1966 (19). It has been found that the protein is widely expressed in various tissues and possesses pleiotropic effects when it is released into the circulation in a nonconventional protein-secretion pathway (20). As a cytosolic chaperon-like protein, MIF has multiple intrinsic enzymatic activities, such as dopachrome/phenylpyruvate tautomerase and thiol protein oxidoreductase activities (21). It has been reported that there was a high positive correlation between MIF (circulating plasma MIF, mononuclear cell MIF mRNA) and BMI (22). In addition, adipocyte MIF mRNA concentration was also positively associated with adipocytes diameter and independently predicted the peripheral insulin action (23) and the expression of MIF was increased in patients with obesity and diabetes (24). Inhibition or knockdown of MIF or its receptor CD74 could improve the glucose tolerance and insulin resistance, even ameliorate hepatic steatosis induced by HFD (25, 26). However, which enzymatic activity of MIF is responsible for affecting HFD-induced inflammation and insulin tolerance in adipose tissues remains unknown.

In the present study, in order to determine the roles of MIF enzymatic activity in HFD-induced obesity, the knock-in transgenic mice with the tautomerase activity-lacking of MIF (MIF^P1G/P1G^) were generated by replacing the proline of N-terminal of the exon 1 with glycine. Our results showed that the serum MIF and the expression of MIF in adipose tissue were increased in HFD-induced obese mice compared with normal mice. The tautomerase -lacking of MIF protected mice from HFD-induced obesity in MIF^P1G/P1G^ mice. In addition, the reduced the inflammation and apoptosis of adipose tissues and the improved the insulin resistance were observed in MIF^P1G/P1G^ mice compared with wild type mice fed with HFD.

## Materials and Methods

### Animals

MIF-P1G knock-in (MIF^P1G/P1G^) transgenic mouse was made by Model Animal Research Centre (Nanjing, China), and the N-terminal proline (Pro1) encoded by exon 1 of MIF was replaced with glycine (P → G) by exchanging the codon CCT with GGC (Pro1 → Gly). The method of generation of MIFP1G/P1G mice was introduced in detail by Gunter Fingerle-Rowson in 2009 and Adamali H in 2012 (27, 28). MIF^P1G/P1G^ mice were a kind gift from GlaxoSmithKline Pharmaceutical Research and Development Co., Ltd (Shanghai, China). MIF^P1G/P1G^ male mice and their littermate wild type male mice with an age of four months were used in this study.

Mice were kept in the animal facility under standard conditions (humidity 50 ± 15%, temperature 22 ± 2°C, 12/12 light-dark cycle) and food and water were provided *ad libitum*. Mice were randomly divided into four group (*n* = 6 per group): (1) WT ND group: wild type mice were fed with normal diet (ND, Jiangsu Cooperative Pharmaceutical Biological Engineering Co., Ltd.); (2) MIF^P1G/P1G^ ND group: MIF^P1G/P1G^ mice were fed with ND; (3) WT HFD group: wild type mice were fed with HFD (60% fat, D12492; Research Diets, New Brunswick, NJ); (4) MIF^P1G/P1G^ HFD group: MIF^P1G/P1G^ mice were fed with HFD. Mice were fed with HFD for 12 weeks for inducing obesity and further experiments.

All animals were treated in accordance with the Guide for the Care and Use of Laboratory Animals of Nanchang University. The experimental protocols were approved by the Ethics Committee of Nanchang University. All experiments were conformed to international guidelines on the ethical use of animals.

### Histological Analysis

Adipose tissues were isolated after euthanasia, fixed overnight in 4% formalin and embedded in paraffin blocks. For Hematoxylin-Eosin (H&E) staining, tissues sections of 5 micron thickness were obtained on poly-l-lysine (Sigma) coated slides using microtome and were stained with hematoxylin and eosin (H&E) according to standard protocol. Representative photomicrographs of H&E stained slide were captured with light microscopy.

### Measurement of Serum MIF

At the end of 12 weeks, the mice in each group were weighed and the blood samples were collected by cardiac puncture under anesthesia after overnight fasting. Trunk blood was collected into EDTA-containing tubes. Plasma was prepared by low-speed centrifugation (3,000 g, 15 min) and was stored at −80°C for subsequent processing. Serum MIF was detected using ELISA Kit for MIF (Cloud Clone Corp. USA) according to the manufacturer's instructions.

### Measurement of F4/80 Immunofluorescence

White adipose tissues (WAT) were isolated from around the epididymis and embedded in paraffin after fixed in 4% paraformaldehyde overnight, dehydrated with ethanol solutions, and transparented with Xylene. Four micron paraffin-embedded WAT sections were deparaffined, rehydrated and endogenous peroxidase quenched with 3% hydrogen peroxide, and microwaved for 15 min (with low heat) in citrate buffer (10 mM sodium citrate, pH 6) to repair antigen, and were then incubated for 60 min in blocking buffer [5% (v/v) goat normal serum (Millipore Bioscience Research Reagents) in PBS containing 0.3% (v/v) Triton X-100 (Sigma)] at room temperature (RT) and subsequently stained with anti-mouse F4/80 (Abcam,ab6640,1:100) antibody for overnight at 4°C. After washing the slides 3 times with 1% BSA in PBS, sections were treated with secondary antibody (alexa fluor 488 conjugated anti-Rat IgG, Invitrogen, A-2121, 1:600) for 1 h at RT. The sections were further washed and the nucleus was counter-stained with 4, 6-diamidino-2-phenylindole (DAPI) diluted to a concentration of 2 μg/ml in PBS (1:500) for 5 minutes. Sections were observed under Olympus fluorescence microscope using 20X objective. The Image-Pro software was used for image acquisition.

### Glucose Tolerance Tests (GTT) and Insulin Tolerance Tests (ITT)

The GTT were performed at tenth week after the mice were fed with ND or HFD. Mice were fasted for 16 hours and then injected intraperitoneally with D-glucose (1.5 g/kg body weight). Glucose concentrations were determined using a glucometer (OneTouch Ultra; LifeScan, Inc.) in blood collected from the tail vein at the indicated time points. The ITT were performed at eleventh week after the mice were fed with ND or HFD. 4-hour-fasted mice were injected intraperitoneally with insulin (0.5 U/kg body weight) and tail vein blood glucose was then measured at the indicated times.

### Quantitative Real Time PCR Analysis (qRT-PCR)

Total RNA from WAT was isolated using the TRIzol method (Invitrogen, MA, USA) followed by DNase treatment. RNA concentration was measured by Nano 2000 (Thermo Fisher). Then RNA was reversely transcribed using a High-Capacity cDNA Reverse Transcription Kit (Applied Biosystems) according to manufacturer's instructions and the cDNAs were stored at −80°C until use. The qRT-PCR was performed using the ABI-ViiA7 PCR machine. The expression of genes was normalized by GAPDH and relative expression was calculated using the ΔCt method. Primers used for real-time PCR are shown as follows: GAPDH (F-AGCCAAAAGGGTCATCATCT; R-GGGGCCATCCACAGTCTTCT), TNF-α (F-GTGGAACTGGCAGAAGAGGCA; R-AGAGGGAGGCCATTTGGGAAC), MCP-1 (F-TCACCTGCTGCTACTCATTCAC; R-CCATTCCTTCTTGGGGTCAG), IL-1beta (F-AAGGCTCCGATGAACAA, R-AAGGCATTAGAAACAGTCC), F4/80 (F- CTTTGGCTATGGGCTTCCAGTC, R-GCAAGGAGGACAGAGTTTATCGTG), IL-6 (F-GGAAATCGTGGAAATGAG, R-GCTTAGGCATAACGCACT), IRS-1 (F- GAGAAGAGACTGGCTCGGAAGA, R-GCCTATTCTGCCCAACTCAACT), IRS-2 (F-GGCCCGAACCTCAATAACAA, R- CCGCGCAACACGAAAAAG), PPARγ (F-TGGGAGATTCTCCTGTTGAC, R-AGGTGGAGATGCAGGTTCTA), GLUT4 (F-GCCCCACAGAAGGTGATTGA, R-AGCGTAGTGAGGGTGCCTTGT), Bax (F-AGGATGCGTCCACCAAGAAG, R-CCATATTGCTGTCCAGTTCATCTC), Bcl2 (F-ATGTGTGTGGAGAGCGTCAA, R-AGAGACAGCCAGGAGAAATCA). All primer pairs used were purchased from Sangon Biotech (Shanghai, China).

### Western Blotting Analysis

Adipose tissues were lysed with RIPA buffer (added with 1 mM PMSF) and then centrifuged at 13,000 rpm for 15 min at 4°C. Protein concentration was measured with pierce BCA Protein Assay Kit (Thermo Fisher Scientific, Rockford, USA). Lysates were resolved by 10% SDS-PAGE, transferred to PVDF membrane (Millipore) and probed with indicated antibodies. The bound antibodies ware detected by second HRP-conjugated antibodies for 1 h at room temperature and visualized by ECL system (Fdbio science, Hangzhou, China).

### Statistical Analysis

The results were presented as mean ± SEM. For GTT/ITT studies with multiple time-points, two-way ANOVA with Turkey *post-hoc* test was used to test for differences. Unpaired t-test and one way ANOVA with turkey post-hoc test was used to test two groups. The software we used to measure the area and the fluorescence was Image J. The value of *P* < 0.05 was considered significant. The statistical analysis was performed using Graph Pad Prism software (Version 7.0) and SPSS19.0.

## Results

### Serum MIF Levels and Expressions of MIF in Adipose Tissues Were Increased in HFD-Induced Obese Mice

**Figure 1:**
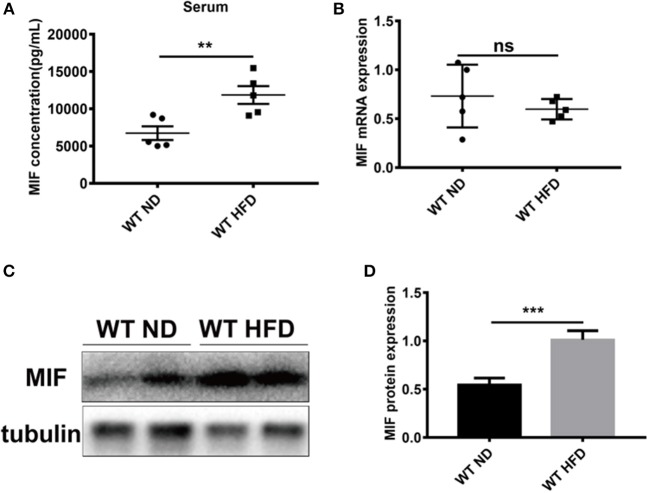
Serum MIF and the expression of MIF in adipose tissue were increased in HFD-treated mice. **(A)** The serum MIF level was determined with MIF ELISA kit in HFD-treated WT mice and ND-treated WT mice. **(B)** The expression of MIF by real-time PCR analysis in adipose tissue from HFD-treated WT mice and ND-treated WT mice. **(C)** The images of MIF protein by Western blot analysis in adipose tissue from HFD-treated WT mice and ND-treated WT mice. **(D)** Quantitative analysis of MIF protein level from western blot bands. Data are shown as mean ± SEM, **p < 0.01 and ***p < 0.001, *n* = 3–5 per group.

To explore the effects of MIF on high fat diet (HFD)-induced obese mice, especially in adipose tissues we first examined the concentrations of serum MIF in HFD-treated mice. As showed in Figures 1A,B, the serum MIF content was significantly increased in HFD group compared with control group, whereas there was no difference in the expressions of MIF mRNA in adipose tissues of both groups. Furthermore, the upregulated expressions of MIF protein in adipose tissues were observed in obese mice compared with control mice (Figures 1C,D). These results suggested that MIF might play a role in adipose tissues of the HFD-induced obese mice.

### MIF^P1G/P1G^ Protected Mice From HFD-Induced Obesity

**Figure 2:**
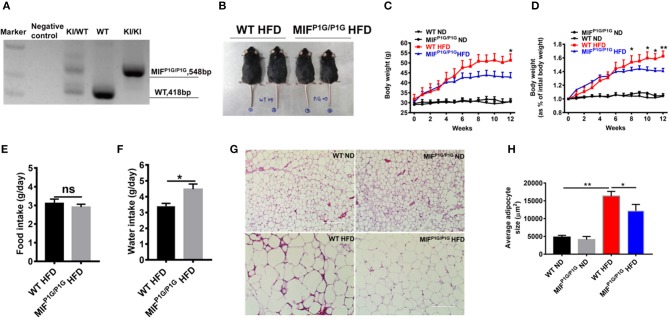
MIF^P1G/P1G^ mice protected mice from HFD-induced obesity. **(A)** The MIF^P1G/P1G^ mouse genotype was identified by RT-PCR. **(B)** An image of the mice fed with high-fat diet (HFD). Four month-old wild-type (WT) and tautomerase-null macrophage migration inhibitory factor (MIF^P1G/P1G^) mice were fed with HFD for 12 weeks. **(C)** The bodyweights of the WT and MIF^P1G/P1G^ mice which were fed with ND or HFD were measured every week. **(D)** The gain curves of bodyweight of mice. The percentage of the bodyweight gain was represented with the initial bodyweight of the mice as a control. **(E)** The food intake between the two groups under HFD. **(F)** The water intake between the two groups under HFD. **(G)** The morphological analysis of Hematoxylin and eosin staining in adipose tissue from the two group mice under ND or HFD. **(H)** The measurement of adipocyte cross-sectional surface area from the two group mice under ND or HFD. Data are shown as mean ± SEM, *p < 0.05 and **p < 0.01, *n* = 5–6 per group.

Although it has been reported that MIF deficiency alleviated HFD-induced obesity, the role of the tautomerase of MIF in HFD-induced obesity was not explored. To investigate the effects of MIF tautomerase activity on obesity, the MIF-P1G knock-in (MIF^P1G/P1G^) mice with lacking of the tautomerase activities of MIF were prepared and the influence of the mutant mice on the HFD-induced obesity were also evaluated. As shown in Figure 2A, the genotype of the mutant mice was verified by PCR. The selective knockout of tautomerase activity of MIF was validated in vitro (Figure S1A). And the results showed that the body weight of the MIF^P1G/P1G^ mice were lighter than WT mice fed with HFD (Figure 2B). Moreover, the bodyweights were significantly reduced in MIF^P1G/P1G^ mice fed with HFD compared with control group, and there were no differences in both groups mice fed with ND (Figures 2C,D). In addition, we observed that the weights of perirenal fat, epididymal fat and liver were decreased in MIFP1G/P1G mice compared with WT mice under HFD (Figures S2B,D). Though we did not examine the changes in muscle, the expression of PPARα was not changed between two groups under HFD (Figure S2C). We also observed that there was no difference in food intake between the two groups (Figure 2E), but the water intake was increased in MIF^P1G/P1G^ mice compared with WT mice (Figure 2F). In addition, H&E staining results showed that the size of adipocyte was smaller in MIF^P1G/P1G^ mice compared with WT mice fed with HFD, whereas there was no significant difference between two groups fed with ND (Figure 2G). And the quantitative measurement of adipocyte cross-sectional surface area was consistent with the HE staining results (Figure 2H). Taken together, our results indicated that tautomerase activity-lacking of MIF alleviated HFD-induced obesity.

### The Inflammation of the Adipose Tissues Was Attenuated in MIF^P1G/P1G^ Mice Fed With HFD

**Figure 3:**
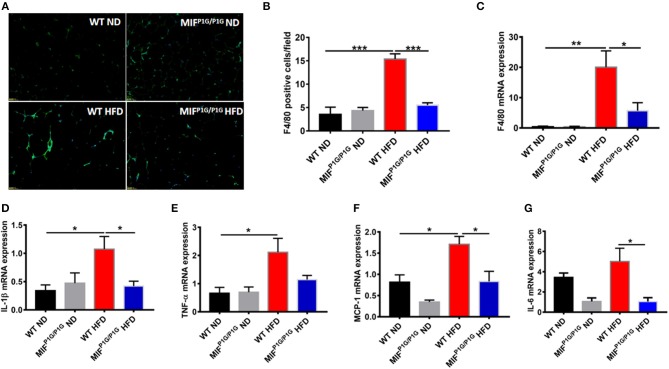
MIF^P1G/P1G^ mice reduced adipose tissue inflammation under HFD. **(A)** The expression of macrophage marker F4/80 in adipose tissue from the two group mice under ND or HFD was examined with immunofluorescence. **(B)** The quantification of the fluorescence image. **(C)** The expression of F4/80 by real-time PCR analysis in adipose tissue from the two group mice. **(D–G)** The expression of pro-inflammatory including IL-1β, TNFα, MCP1, and IL-6 by real-time PCR analysis in adipose tissue from the two group mice. Data are shown as mean ± SEM, *p < 0.05, **p < 0.01, and ***p < 0.001, *n* = 5–6 per group.

Mild inflammation plays an important role in obesity. To explore the impact of the tautomerase activity-lacking of MIF on inflammation in HFD-induced obese mice, the expression of F4/80, the macrophage maker in adipose tissue, was examined by immunofluorescence. Our results showed that the expression of F4/80 was increased in HFD-treated WT mice compared with ND-treated WT mice, while MIF^P1G/P1G^ significantly decreased the expression of F4/80 in mice fed with HFD (Figure 3A). The determination of the F4/80-positive cells that represented macrophage infiltration did further confirm the results of the immunofluorescence image (Figure 3B). In addition, the expression of F4/80 mRNA was also decreased in adipose tissue of MIF^P1G/P1G^ mice compared with WT mice fed HFD (Figure 3C). Furthermore, the expressions of pro-inflammatory cytokines such as IL-1β, TNFα, MCP1, and IL-6 were upregulated in the adipose tissues of the HFD-treated WT mice compared with ND-treated WT mice, whereas MIF^P1G/P1G^ mice significantly decreased the elevations in the mice fed with HFD (Figures 3D–G). To further investigate the effects of the lack of the MIF tautomerase activity on HFD-induced obesity, we detected the expressions of the genes related with lipid synthesis (Srebp-1c, Fans, SCD-1), lipolysis (ATGL, HSL, G0S2), fatty acid oxidation (PPARα, CPT-1α, ACOX1) and adiposeătissueăbrowning (UCP1, PGC-1α, adiponectin, Elov13, Dio2 and Prdm16) in white adipose tissues. As showed in Figures S1B–E, there were no significant changes between MIFP1G/P1G HFD and WT HFD group, suggesting that lack of MIF tautomerase activity had little or no effects on WAT lipid metabolite. All these results indicated that MIF^P1G/P1G^ reduced inflammation induced by HFD.

### HFD-Induced Insulin Resistance Was Ameliorated in MIF^P1G/P1G^ Mice

**Figure 4:**
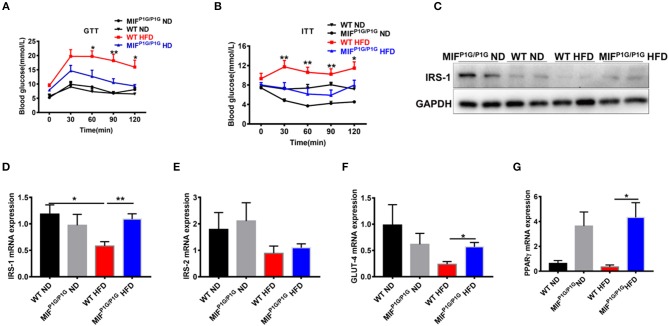
MIF ^P1G/P1G^ mice ameliorated HFD-induced insulin resistance. **(A)** Glucose tolerance test (GTT) was examined in WT and MIF^P1G/P1G^ mice fed ND or HFD for 10 weeks after 16 h fasted. **(B)** Insulin tolerance test (ITT) was examined in WT and MIF^P1G/P1G^ mice fed ND or HFD for 11 weeks after 4 h fasted. **(C)** The images of IRS1 protein by Western blot analysis in adipose tissue from WT and MIF^P1G/P1G^ mice fed ND or HFD. **(D–G)** The expression of IRS1, IRS-2, GLUT4, and PPARγ by real-time PCR analysis in adipose tissue from the two group mice. Data are shown as mean ± SEM, *p < 0.05, **p < 0.01, *n* = 4–5 per group.

Chronic inflammation is an important factor in obesity-related insulin resistance. In order to explorer the roles of MIF tautomerase activity-lacking in HFD-induced insulin resistance, we first examined the glucose intolerance and insulin resistance using GTT and ITT assays. The results showed that the glucose intolerance and insulin resistance were significantly improved in MIF^P1G/P1G^ mice fed with HFD compared with control mice (Figures 4A,B). In addition, the expression of IRS-1 was decreased in the adipose tissues in HFD-treated WT mice compared with ND-treated mice, while its expression was increased in MIF^P1G/P1G^ group compared with control group under HFD (Figures 4C,D). But there were no significant differences in the expression of IRS-2 between WT and MIF^P1G/P1G^ mice (Figure 4E). Moreover, our results also showed that the expression of GLUT4 was upregulated in adipose tissue from MIF^P1G/P1G^ mice fed with HFD (Figure 4F). Study showed that PPARγ is an important target for diabetes drugs, and its agonist can improve diabetes. In our study, we also found the expressions of PPARγ were significantly increased in MIF^P1G/P1G^ group under both normal diet or HFD conditions compared with control group (Figure 4G). Taken together, our results demonstrated that MIF^P1G/P1G^ ameliorated HFD-induced insulin resistance via affecting insulin signaling pathway.

### Tautomerase Activity-Lacking of MIF Attenuated the HFD-Induced Apoptosis of Adipocytes in MIF^P1G/P1G^ Mice

**Figure 5:**
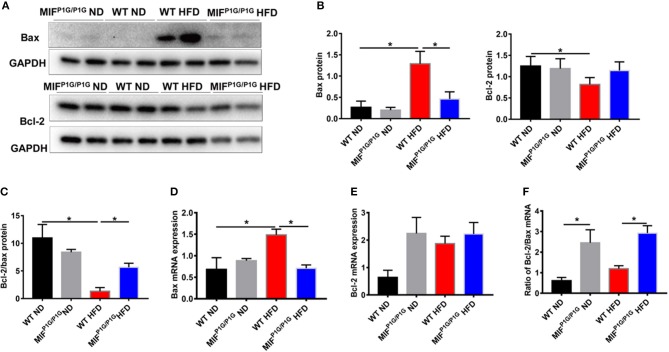
MIF ^P1G/P1G^ mice ameliorated adipocyte apoptosis under HFD. **(A)** The images of Bax and Bcl2 protein by Western blot analysis in adipose tissue from WT and MIF^P1G/P1G^ mice fed ND or HFD. **(B)** The quantitative analysis of the protein level of the Bax and Bcl2 was determined in adipose tissue from WT and MIF^P1G/P1G^ mice fed ND or HFD. **(C)** The ratio of Bcl2/bax in protein level was quantitatively determined in adipose tissue from the two group mice. **(D–E)** The expression of Bax and Bcl2 by real-time PCR analysis in adipose tissue from the two group mice. **(F)** The ratio of Bcl2/Bax was quantitatively determined by qPCR in adipose tissue from the two group mice. Data are shown as mean ± SEM, *p < 0.05, *n* = 4–5 per group.

It has been reported that obesity was associated with cell apoptosis. Next, we explored the effects of tautomerase activity-lacking of MIF on adipocyte apoptosis in HFD-induced obese MIF^P1G/P1G^ mice. The results showed that the expression of pro-apoptotic gene Bax was upregulated in adipose tissue from HFD-treated WT mice compared with that from ND-treated WT mice. But its expression was reduced in MIF^P1G/P1G^ mice under HFD (Figures 5A,B). Moreover, we found that the expression of anti-apoptotic gene Bcl2 was decreased in adipose tissue from HFD-treated WT mice compared with that from ND-treated WT mice. But there was no significant difference between the MIF^P1G/P1G^ and WT groups under HFD (Figures 5A,B,E). Moreover, we found the ratio of Bcl-2/bax in protein level was reduced in WT mice under HFD compared with ND, whereas it was increased in MIF^P1G/P1G^ mice compared with WT mice on HFD (Figure 5C). The expression of Bax in mRNA level was consistent with the protein level (Figure 5D). As showed in Figure 5F, the ratio of Bcl2/bax in mRNA level was significantly increased in the MIF^P1G/P1G^ mice compared with control group under HFD. Furthermore, we detected the expression of activated caspase 3 by immunohistochemistry. As showed in Figure S2A, the number of cleaved caspase 3-positive cells in white tissue was obviously increased in WT mice under HFD compared with normal diet, whereas it was reduced in MIFP1G/P1G mice compared with WT mice under HFD. These findings suggested that the reduced apoptosis may play a role in MIF^P1G/P1G^-mediated attenuation of HFD-induced obesity.

## Discussion

Several studies demonstrated that MIF played an important role in the development of obesity and the associated diseases including insulin resistance, T2D, cardiovascular disease (CVD) and NAFLD (25, 29, 30). Obviously, obesity is an important risk factor for many diseases. Studies showed that there was a positive association between obesity and serum circulating MIF levels (31, 32). In the present study, we observed that the serum circulating MIF levels and the expression of MIF protein in adipose tissues were increased in HFD mice although there was no significant difference in the RNA level. These results suggested that MIF might play a role in obesity.

MIF is a cytokine which is widely expressed in various tissues. It has been reported that MIF has a pro-inflammatory role through its two key enzymatic activities, tautomerase, and oxidoreductase (33, 34). Recently, some literatures showed that MIF was also involved in energy metabolism. Researches showed that MIF-deficiency reduced chronic inflammation in white adipose tissue and impaired the development of insulin resistance (25). There are also reports that MIF deficiency promoted adiposity in fructose-fed mice (35). Many studies have focused on the effects of MIF knockout on metabolism. Few studies have investigated the effects of MIF enzymatic activity on metabolism. In this study, we focused our attention on effects of tautomerase activity-lacking of MIF on obesity induced by HFD. The results showed that the bodyweight was reduced in MIF^P1G/P1G^ mice under HFD. In addition, the size of adipocyte was smaller in MIF^P1G/P1G^ mice compared with control mice fed with HFD. Interestingly, we also adopted another enzymatic active site (thiol-protein oxidoreductase null), named as MIF^C60S/C60S^ mice. The unpublished data showed that there was no significant difference in the mice when fed with HFD. These results further demonstrated that the tautomerase of MIF played an important role in HFD-induced obesity.

It has been reported that the excessive food and energy intake induce chronic low-grade activation of inflammation of adipose tissue with macrophage infiltration, adipocyte hypertrophy and increasing cytokines (36). In the early stage of obesity, chemotactic monocyte chemoattractant protein-1 (MCP-1)/C-C chemokine receptor 2 (CCR2) are secreted by hypertrophic adipocyte, in which the cytokines promote the accumulation of macrophage in obese adipose tissue. Studies showed that the secreted MIF can directly facilitate M1 macrophage polarization through CD74 (26, 36). In the advanced stage of obesity, M1 macrophage-derived MIF further exacerbates both adipocyte inflammation and macrophage polarization in a positive feedback loop, resulting in the exaggeration of adipose tissue inflammation and insulin resistance (10, 26). In addition, the increased macrophage infiltration elevated the levels of pro-inflammatory cytokines including MIF, IL-1β, MCP-1, and TNF-α in WAT (37). In our study, the macrophage infiltration and the expressions of pro-inflammatory cytokines were increased after HFD treatment, whereas MIF^P1G/P1G^ alleviated HFD-induced inflammation. These results indicated that the tautomerase of MIF played important roles in HFD-induced obesity via affecting inflammation.

A few studies showed that obesity was closely related to the occurrence of diabetes mellitus, especially type 2 diabetes (38). It was reported that the accumulation of abdominal fat is associated with insulin resistance (39). During obesity, several mechanisms played roles in leading to insulin resistance, among that white adipose tissue inflammation was vital. The altered adipokines and pro-inflammatory cytokines such as IL-6, TNF-α, and MCP-1 which secreted from adipose tissue may directly or indirectly caused insulin resistance through impairing insulin signaling or activating pro-inflammatory pathways (14). In the present study, we found the inflammation was decreased in adipose tissue from MIF^P1G/P1G^ mice under HFD. To further identify whether the reduced inflammation could be able to improve insulin resistance in MIF^P1G/P1G^ mice under HFD, the GTT and ITT assays were first performed in our study. Our results showed that the glucose intolerance and insulin resistance were improved in MIF^P1G/P1G^ mice under HFD via enhancing insulin signaling. In addition, it was reported that PPARγ was an important receptor in the treatment of diabetes, and its activation reduced hyperglycemia by increasing sensitivity to peripheral insulin (40). We also found the expression of PPARγ was increased in adipose tissue from MIF^P1G/P1G^ mice. Taken together, these findings indicated that the tautomerase activity-lacking of MIF improved insulin resistance.

It has been reported that a series of factors related to apoptosis including leptin and TNF-α were significantly higher in serum from obese patients than control. In addition, it was reported that TNFα had been shown to induce adipocyte apoptosis *in vivo* and *in vitro* (41, 42). Our results showed that adipocyte apoptosis was increased in HFD-treated mice compared with ND-treated mice, manifested by the increased expression of pro-apoptotic gene Bax. While MIF^P1G/P1G^ significantly decreased its expression. Moreover, we found the ratio of Bcl2/Bax was increased in MIF^P1G/P1G^ mice. All these results suggested that MIF^P1G/P1G^ could reduce adipocyte apoptosis *in vivo* under HFD circumstance. Although we observed the alternations in MIF^P1G/P1G^ mice with lacking of the tautomerase activities of MIF under HFD, there were still limitations in our research. For example, the specific mechanisms of the body-weight loss in genetic manipulation of MIF still need to be further explored using primary adipocytes and macrophages of MIF^P1G/P1G^ *in vitro*. In addition, the relationship between the reduced inflammation and insulin resistance and the reduced weight gain in MIF^P1G/P1G^ mice under HFD also need to be further investigated.

## Conclusion

Our study demonstrated that the tautomerase activity-lacking of MIF significantly alleviated HFD-induced obesity in mice, in which the alleviation may be related to the inhibition of the HFD-induced inflammation and insulin resistance in adipose tissues by the lack of MIF tautomerase activity. However, the accurate mechanisms remain to be investigated.

## Data Availability Statement

The raw data supporting the conclusions of this article will be made available by the authors, without undue reservation, to any qualified researcher.

## Ethics Statement

All the experimental procedures were approved by Nanchang University Institutional Animal Research Committee and were carried out in accordance with Jiangxi Province Laboratory Animal Care Guidelines for the use of animals in research.

## Author Contributions

H-BX and L-FW designed the experiments. The experimental procedures were performed by Y-HL. KW, L-LZ, S-KL, QC, and QL participated in the data analysis. LD, TC, XW, and K-YD gave experimental guidance. Y-HL and L-FW drafted the manuscript. H-BX revised the manuscript.

### Conflict of Interest

The authors declare that the research was conducted in the absence of any commercial or financial relationships that could be construed as a potential conflict of interest.

## Supplementary Material

Figure S1 The expressions of lipid metabolism related genes in MIF^P1G/P1G^ mice and WT mice. **(A)** The selective knockout of tautomerase activity of MIF was validated *in vitro*. **(B)** The expressions of lipid synthesis related genes. **(C)** The expressions of lipolysis related genes. **(D)** The expressions of fatty acid oxidation related genes mRNA expression. **(E)** The expressions of adipose tissue browning related genes mRNA expression. Data are shown as mean ± SEM, ^*^*p* < 0.05. *n* = 4–6 per group.

Figure S2 MIF^P1G/P1G^ mice reduced WAT apoptosis and the weight of perirenal fat, epididymal fat, and liver tissue under HFD. **(A)** Immunohistochemical analysis of cleaved-Caspase-3 staining in WAT. **(B)** The weight of perirenal fat, epididymal fat, and subcutaneous fat. **(C)** The expression of PPARα in muscle between two groups under ND and HFD. **(D)** The weight of liver tissue between two groups under HFD. Data are shown as mean ± SEM, ^*^*p* < 0.05, *n* = 5 per group.
