# Supplementary figures and images for: Tautomerase Activity-Lacking of the Macrophage Migration Inhibitory Factor Alleviates the Inflammation and Insulin Tolerance in High Fat Diet-Induced Obese Mice
Source: Front Endocrinol (Lausanne). 2020 Mar 20;11:134. doi: 10.3389/fendo.2020.00134 (PMC7098947; doi:10.3389/fendo.2020.00134)

Figure S1

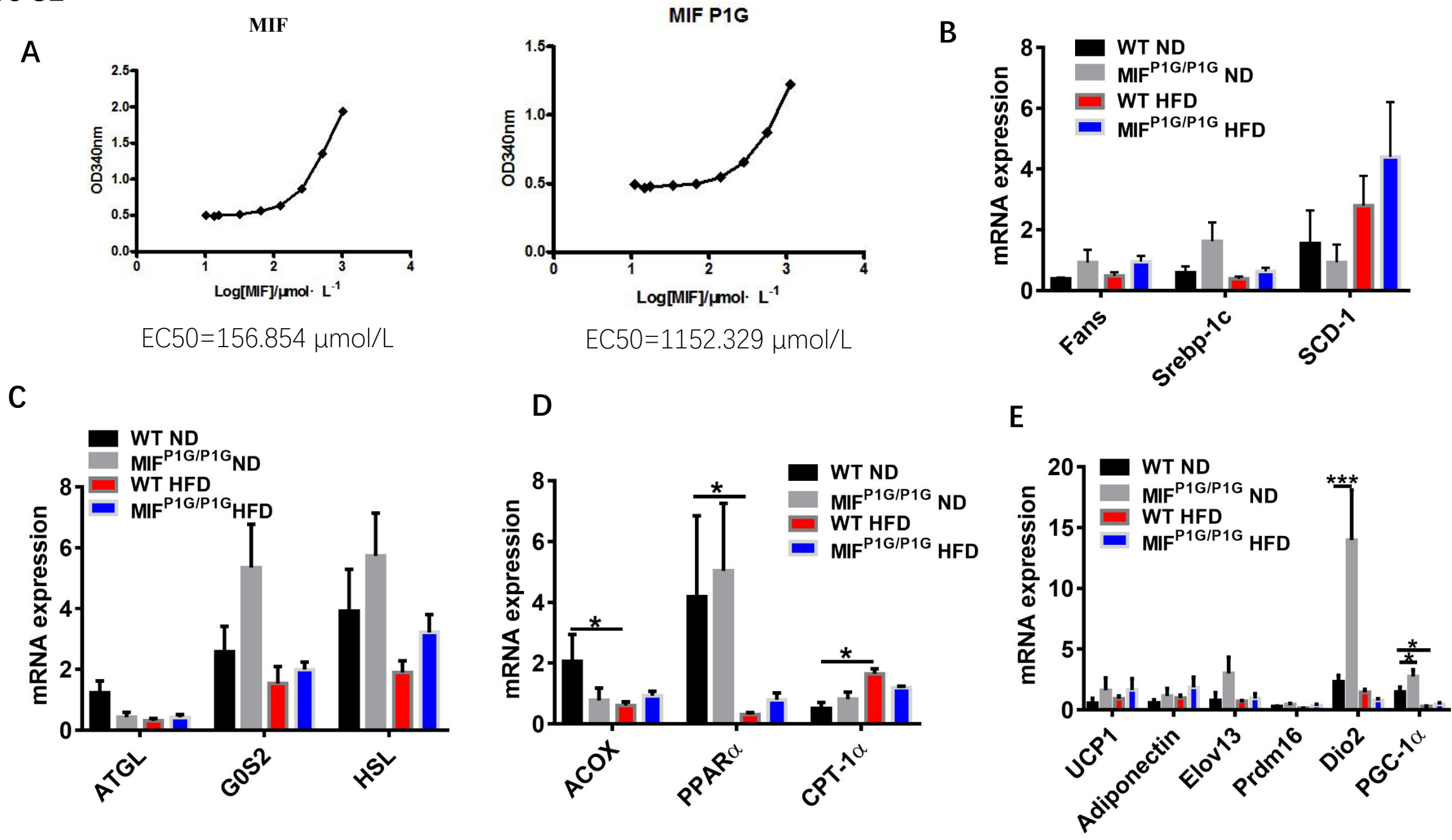

Figure S2

A

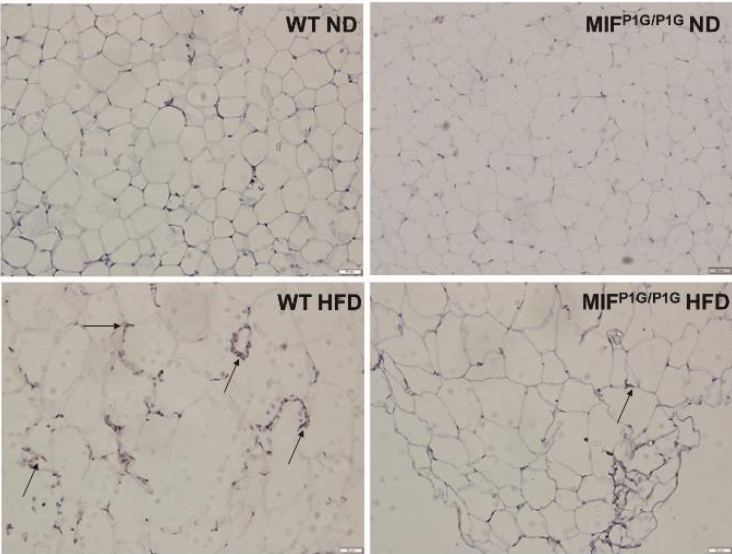

B

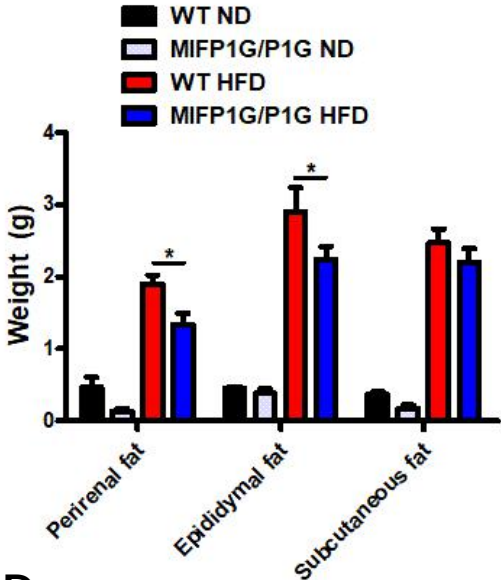

C

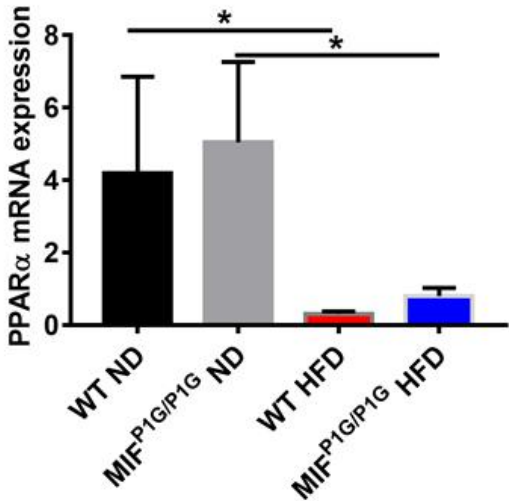

D

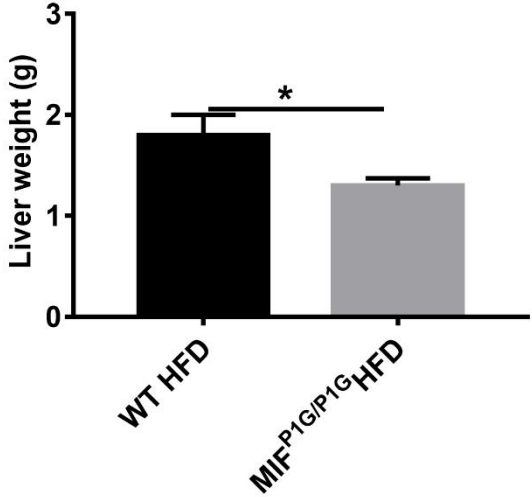

Supplement: Figure S1 — The expressions of lipid metabolism related genes in MIFP1G/P1G mice and WT mice. (A) The selective knockout of tautomerase activity of MIF was validated in vitro. (B) The expressions of lipid synthesis related genes. (C) The expressions of lipolysis related genes. (D) The expressions of fatty acid oxidation related genes mRNA expression. (E) The expressions of adipose tissue browning related genes mRNA expression. Data are shown as mean ± SEM, *p < 0.05. n = 4–6 per group. [file Data_Sheet_1.PDF]
